# Rapid Detection of Peste Des Petits Ruminants via Multienzyme Isothermal and Lateral Flow Dipstick Combination Assay Based on N Gene

**DOI:** 10.3390/vetsci13010110

**Published:** 2026-01-22

**Authors:** Jiamin Zhou, Jiao Xu, Jiani Li, Jiarong Yu, Yingli Wang, Jingyue Bao

**Affiliations:** 1 China Animal Health and Epidemiology Center, Qingdao 266000, China; zjm2020925@163.com (J.Z.); xujiao@cahec.cn (J.X.); lijiani07300@163.com (J.L.); yujiarong@cahec.cn (J.Y.); wangyingli@cahec.cn (Y.W.); 2 College of Veterinary Medicine, Gansu Agricultural University, Lanzhou 730000, China; 3 College of Veterinary Medicine, Qingdao Agricultural University, Qingdao 266000, China

**Keywords:** peste des petits ruminants, multienzyme isothermal rapid amplification, rapid detection, diagnosis

## Abstract

In this study, a multienzyme isothermal and lateral flow dipstick combination assay for PPRV detection was established, the designed primers and probes targeting the N gene were screened and optimized, and analytical sensitivity, specificity, and repeatability of developed method were systematically evaluated. The experimental results demonstrated that this method is easy to operate, can complete detection within 30 min at 42 °C, and is capable of detecting all lineages of peste des petits ruminants virus (PPRV) without cross-reactivity with other viruses. The limit of detection could reach 10 copies/μL. Repeatability validation showed that the coefficients of variation (CV) for both intra-assay and inter-assay experiments were below 3.0%. The positive detection rate for clinical samples could reach 100%. The test results are visually interpretable via fluorescence and lateral flow strips. In conclusion, this method exhibits high analytical sensitivity, specificity, and excellent repeatability, enabling rapid diagnosis of peste des petits ruminants (PPR).

## Simple Summary

In this study, we established a visual rapid diagnostic method for peste des petits ruminants and validated its sensitivity, specificity, and repeatability. The results demonstrate that this method can rapidly and accurately detect the peste des petits ruminants virus with sensitivity comparable to current mainstream detection methods. Moreover, it requires less operational time, demands lower expertise from operators, and can be performed even under extreme conditions without the need for a laboratory environment. The method shows no cross-reactivity with other common animal diseases, exhibits good repeatability, and produces consistent and highly accurate results for clinical samples compared to other established methods. Additionally, the detection outcomes can be presented in multiple visual formats and are easy to interpret.

## 1. Introduction

Peste des petits ruminants (PPR) is a highly contagious infectious disease caused by the peste des petits ruminants virus (PPRV). PPRV belongs to the family Paramyxoviridae, subfamily Orthoparamyxovirinae, and genus Morbilliviruses species Morbillivirus caprinae and has a linear negative-stranded RNA genome [1]. It primarily infects goats, sheep, and wild small ruminants, with clinical symptoms mainly including fever, increased oral and nasal discharge, and diarrhea [2]. PPR is a notifiable animal disease designated by the World Organisation for Animal Health (WOAH) and is classified as a Class I animal disease in China. It was first discovered in Africa, where it has been endemic on the African continent for a long time, and has gradually spread to Asia. In 2018, it entered Europe, specifically Bulgaria, but its impact was relatively limited. In the summer of 2024, the disease resurged in Europe, with Greece, Romania, and Hungary and other European countries successively reporting outbreaks of PPR [3]. As of late 2025, the outbreaks in these countries had not yet concluded, marking a further expansion of the PPR epidemic range. PPR causes huge economic losses to the animal breeding industry each year. Consequently, building on the successful experience of eradicating rinderpest, WOAH has targeted PPR for global control and eradication by 2030.

In 2007, China firstly reported a PPR outbreak in Tibet; following the outbreak, emergency measures such as vaccination, movement controls, and compulsory culling were promptly implemented in the affected areas. Due to timely intervention, the outbreak was quickly contained and did not spread to other provinces. In 2013, the virus re-emerged in Xinjiang. Although China implemented timely response measures, as a major province for live sheep transportation, large numbers of live sheep transported nationwide via a convenient transportation network from Xinjiang. Consequently, the outbreak quickly spread to other provinces, leading to a sharp increase in reported cases. It was not until the second half of 2014 that the epidemic situation gradually stabilized, though sporadic cases continued to occur domestically.

PPRV is a single-stranded, negative-sense, non-segmented RNA virus. Its genome, from 3′ to 5′, consists of the N-P-M-F-H-L genes, encoding six structural proteins and two non-structural proteins. PPRV has only one serotype. However, based on the genetic evolutionary characteristics of the N or F gene, the virus can be divided into four lineages [4,5]. Lineages I and II are primarily prevalent in Africa, while Lineage III is distributed in some countries in the Middle East. Lineage IV has a broader prevalence, being found in Africa, Asia, and Europe. Recent studies indicate that it is replacing other lineages to become the dominant epidemic lineage in Africa [6,7,8,9]; nevertheless, based on the global epidemic situation, detection methods for viruses of all lineages remain essential.

The non-specific clinical symptoms of PPR necessitate laboratory testing for definitive diagnosis. For the detection of peste des petits ruminants (PPR), multiple methods have been developed, among which RT-qPCR is the most widely used; however, this method requires specialized personnel and equipment, and a complete reaction procedure may require 2 to 3 h to complete, making it unsuitable for rapid testing. The recombinase polymerase amplification (RPA) assay has been proposed for the rapid identification of various pathogens under field conditions with limited resources such as RT-LAMP. It is an isothermal nucleic acid amplification method that, by employing an enzymatic primer–protein binding process, can complete target gene amplification within 30 min at a relatively low temperature [10]. As a new RPA method, multienzyme isothermal rapid amplification (MIRA) is an emerging isothermal nucleic acid amplification technique analogous to recombinase polymerase amplification. The method employs four core proteins-recombinase, DNA helicase, single-stranded DNA-binding protein, and DNA polymerase to amplify target genes under isothermal conditions [11]. MIRA can be performed at 25 to 42 °C within 5 to 30 min, requiring only a water bath to maintain a constant temperature. Furthermore, when combined with colloidal gold-based lateral flow detection (MIRA-LFD), results can be visually interpreted using a test strip [12]. In recent years, it has been widely applied in the surveillance of animal viral diseases as well as important human viral diseases, which all showed high sensitivity. However, MIRA has not yet been applied to the detection of PPRV. In this study, we established the rapid MIRA-based detection systems (including fluorescence- and colloidal gold-based formats) for PPRV identification.

## 2. Materials and Methods

### 2.1. Primer and Probe Design

The complete genomic sequences of all four lineages of PPRV strains were obtained from GenBank (Table S1) and aligned using SnapGene software (v8.1, GSL Biotech LLC, Boston, MA, USA). Primers and probes for the recombinase-aided amplification (MIRA) assay were designed based on the conserved region of the PPRV nucleoprotein (N) gene. Based on preliminary optimization and screening, the primers and probes used in this experiment have been determined. The forward primer sequence was 5′-CAGTCCGGGTTGACCTTTGCATCACGTGGTGCTGA-3′, and the reverse primer sequence was 5′-TGTGTYTATTTAACCCACCTTCTCA-3′. The probe sequence was 5′-AAAGGATCAACTGGTTTGAGAACAGAGAAA[FAM-dT]A[THF][BHQ1-dT]AGACATAGARGTGCA-3′-C3-spacer. To combine lateral flow dipstick (LFD) detection with the MIRA assay, the primers and probes were modified accordingly. The forward primer remained 5′-CAGTCCGGGTTGACCTTTGCATCACGTGGTGCTGA-3′. The reverse primer was labeled with biotin at the 5′-end: 5′-Biotin-TGTGTYTATTTAACCCACCTTCTCA-3′. The probe was modified as 5′-FAM-AAAGGATCAACTGGTTTGAGAACAGAGAAAA[THF]TAGACATAGARGTGCA-3′-C3-spacer.

### 2.2. Viruses and Plasmids

Strains of Goat pox virus, Orf virus, and Foot-and-mouth disease virus were obtained from the National Reference Laboratory for Peste des Petits Ruminants (China Animal Health and Epidemiology Center, Qingdao, China). Plasmids of the four PPRV strains used in this study were constructed and verified by Sangon Biotech (Sangon Biotech (Shanghai) Co., Ltd., Shanghai, China) Co., Ltd. The plasmid copy number (copies·μL^−1^) was calculated according to the following formula: 6.02 × 10^23^ × (plasmid concentration (ng·μL^−1^/[plasmid length (bp) × 10^9^ × 660]). A 10-fold serial dilution series, ranging from 10^5^ to 10^0^ copies·μL^−1^, was prepared and stored at −20 °C for subsequent assays of analytical sensitivity, reproducibility, and specificity.

### 2.3. Establishment of the Reaction

MIRA detection utilizes the DNA Isothermal Rapid Amplification Kit (WLE8202KIT, AMP-Future Biotech Co., Ltd., Changzhou, China) and RNA Isothermal Rapid Amplification Kit (WLRE8208KIT, AMP-Future Biotech Co., Ltd., Changzhou, China). Viral RNA and DNA were extracted beforehand using RNA/DNA extraction kits (DP422, TIANGEN Biotech Co., Ltd., Beijing, China). The reaction system comprises 14.7 µL Buffer A, 4.25 µL RNase-free ddH_2_O, 2.5 µL template, 0.3 µL 10 µmol·L^−1^ probe, and 1 µL each of 10 µmol·L^−1^ forward and reverse primers, placed in a reaction tube containing lyophilized enzyme powder. Next, add 1.25 µL of Buffer B to achieve a total volume of 25 µL. Invert the reaction tube 10 to 15 times to mix thoroughly, then immediately place it in a constant-temperature fluorescence detector (WL-16-III, AMP-Future Biotech Co., Ltd., Changzhou, China). Collect fluorescence signals every 30 s at 42 °C for 30 min. Finally, observe the fluorescence of the reaction tube under blue light.

MIRA-LFD detection was performed using the RNA Isothermal Rapid Amplification Kit (WLRN8209KIT, AMP-Future Biotech Co., Ltd., Changzhou, China) in conjunction with nucleic acid detection test strips (WLFS8204KIT, AMP-Future Biotech Co., Ltd., Changzhou, China). Upon completion of the MIRA reaction, the amplification product was diluted at a 1:20 ratio with RNase-free ddH_2_O. A volume of 80 µL of the diluted product was applied to the sample port of the test strip. Results were documented and interpreted within 5 min.

### 2.4. Analytical Sensitivity and Repeatability Tests

The analytical sensitivity of the MIRA assay was determined using a PPRV plasmid constructed and stored prior to this experiment. The plasmid DNA was serially diluted tenfold from 10^5^ to 10^0^ copies·μL^−1^ using plasmid DNA storage buffer, with ddH_2_O serving as a negative control for analysis. To assess the stability and reliability of the established detection method, the previously constructed PPRV plasmid was employed as the template to evaluate the reproducibility of the MIRA assay. The plasmid was subjected to tenfold serial dilution in plasmid DNA storage buffer, generating concentrations ranging from 10^5^ to 10^1^ copies·μL^−1^. Intra-assay repeatability was determined by calculating the coefficient of variation (CV) from fluorescence values obtained from triplicate measurements of the same sample. For inter-assay repeatability, each plasmid concentration was independently diluted in three separate series and analyzed in triplicate, with the corresponding fluorescence values used to calculate the CV.

### 2.5. Specificity Test

To evaluate the specificity of the newly established MIRA detective method, nucleic acids from PPRV and other important sheep disease viruses including ORFV, GPV, and FMDV were subjected to test. Nuclease-free ddH_2_O was included as a negative control in the testing protocol.

### 2.6. Field Sample Detection

Nucleic acids extracted from 48 collected tissue samples (which had been confirmed as positive or negative using RT-qPCR [13]) were analyzed using MIRA assay. The detection results from the methods were compared to evaluate the accuracy and reliability of the newly established MIRA detective method for PPRV detection in clinical specimens.

## 3. Results

### 3.1. Analytical Sensitivity of the Developed Method

**Figure 1:**
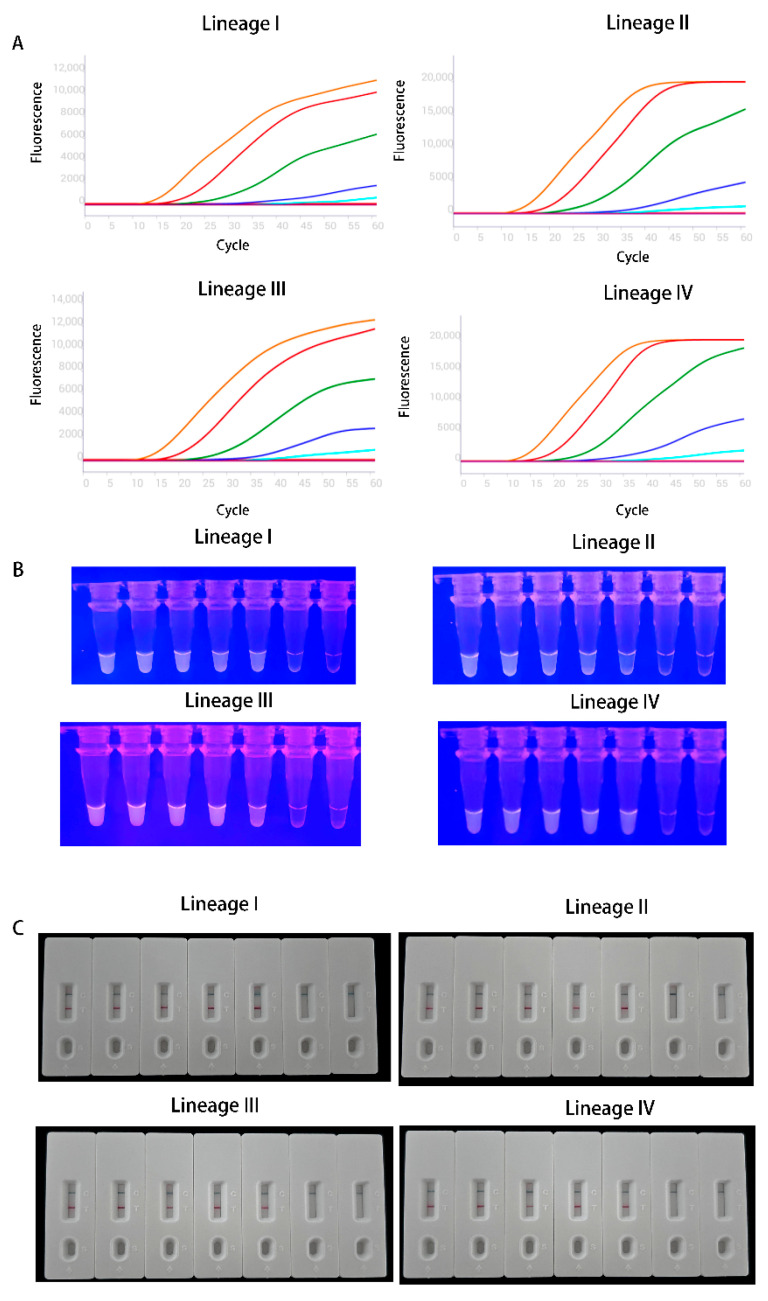
Analytical sensitivity tests of MIRA assay. (**A**) Amplification curves and fluorescence results. The curves display the amplification results of PPRV plasmid DNA from various lineages at concentrations ranging from 10^5^ to 10^0^ copies·μL^−1^ (from left to right). The negative control is RNase-free ddH2O. (**B**) Detective results of MIRA-fluorescence. The detection sensitivity of MIRA-fluorescence to different lineages under blue light observation, with copy numbers of all lineages from left to right in the figure being 10^5^ to 10^0^ copies·μL^−1^ and negative control. (**C**). Detective results of MIRA-LFD. The detection sensitivity of MIRA-LFD to different lineages was observed, with copy numbers of all lineages from left to right in the figure being 10^5^ to 10^0^ copies·μL^−1^ and negative control.

The results are presented in Figure 1, demonstrated that both the MIRA-fluorescence and MIRA-LFD assays could detect 10^1^ copies·μL^−1^ across all PPRV lineages evaluated.

### 3.2. Repeatability of the Developed Method

As summarized in Table 1, all tested lineages showed coefficients of variation (CV) below 3% for both intra- and inter-assay comparisons.

### 3.3. Specificity of the Developed Method

**Figure 2:**
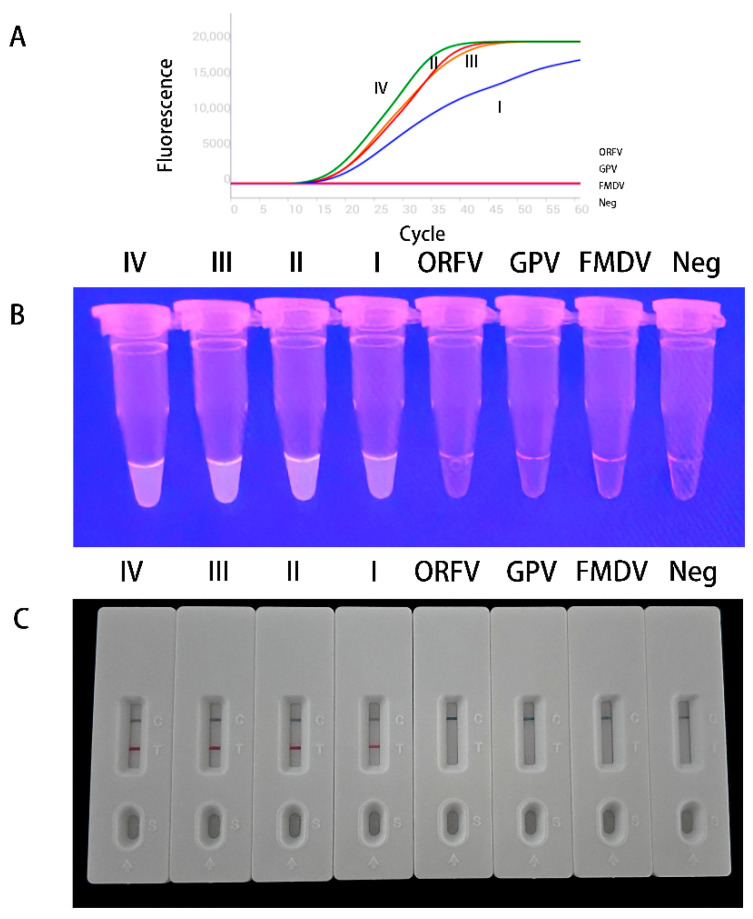
Specificity tests of MIRA assay. (**A**) Amplification curves and fluorescence results. (**B**) Detective results of MIRA-fluorescence. (**C**) Detective results of MIRA-LFD.

As shown in Figure 2, all four PPRV lineages generated fluorescent signals within 30 min, whereas no amplification was observed for ORFV, GPV, or FMDV. Correspondingly, nucleic acid test strips produced positive results for all PPRV lineages but negative results for the non-target viruses.

### 3.4. Detection of Field Samples

**Figure 3:**
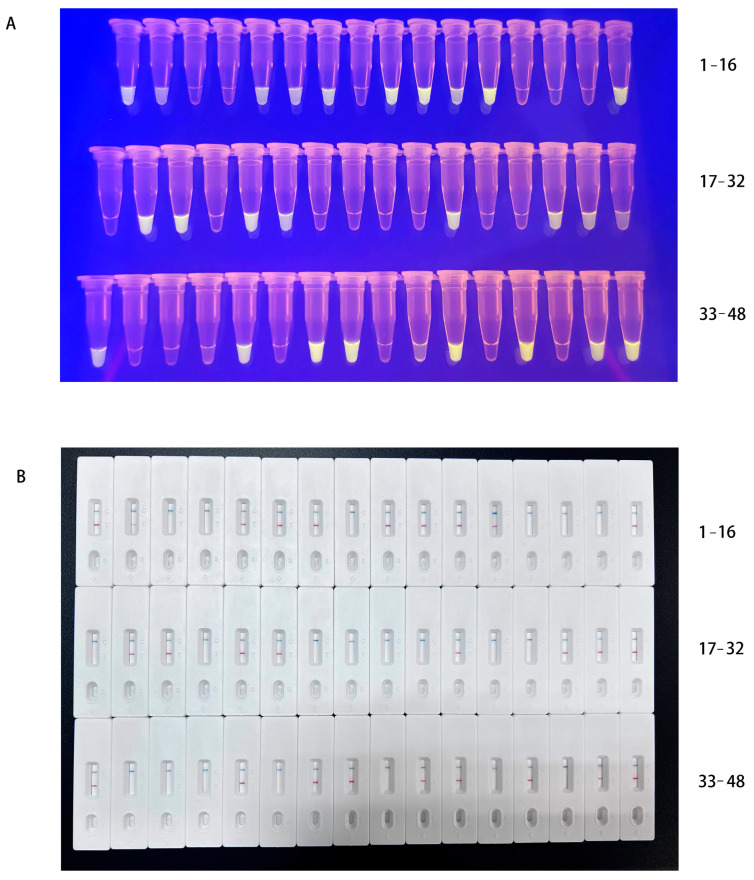
Detective results of clinical samples via (**A**) MIRA-fluorescence and (**B**) MIRA-LFD.

All samples were tested using both fluorescence and lateral flow dipstick. The results are shown in Figure 3, yielding 27 positive results and 21 negative results. The MIRA results were fully consistent with the qPCR results obtained from routine clinical testing for PPRV (Table 2).

## 4. Discussion

Although the World Organisation for Animal Health has set the global goal for eradicating peste des petits ruminants by 2030; since last year, the disease has re-emerged in Europe and continues to spread and expand to this day [14]. Vietnam, in Southeast Asia, has also reported its first outbreak of the disease in 2025. This indicates that the global impact of peste des petits ruminants is further increasing, adding uncertainty to its eradication efforts. At present, there is no specific therapeutic drug for this disease. Although several attenuated vaccines are available with satisfactory protective efficacy, most of them share the characteristic of being thermolabile: their protective capacity diminishes significantly within just a few hours at room temperature [15]. Regions where peste des petits ruminants is endemic, such as Africa and the Middle East, generally experience high average temperatures. Additionally, many developing countries face a shortage of professional veterinary personnel, which collectively poses challenges to the establishment of effective immunization barriers in these areas. Indeed, quickly identifying the source of infection and cutting off transmission routes are crucial for interrupting the spread of the virus. Therefore, establishing reliable detection methods is essential. Although virus isolation is considered as the gold standard for diagnosing peste des petits ruminants, this method is time-consuming, requires highly skilled personnel, and for highly contagious diseases like PPR, it must be conducted in high-level biosafety laboratories (ABSL-3). These factors limit its widespread adoption and application [16,17].

According to the recommendations of the WOAH, RT-qPCR, and ELISA are widely used for etiological and serological confirmation of peste des petits ruminants (PPR), respectively. Since there is currently no vaccine capable of differentiating between natural infection and vaccination-induced antibodies (DIVA vaccine), in countries conducting large-scale vaccination campaigns, ELISA can only be employed to monitor antibody levels and cannot be used for definitive diagnosis of PPR.

Researchers have developed multiple RT-qPCR detection methods targeting the relatively conserved N and F genes of PPRV. Batten and Flannery developed a rapid RT-qPCR assay targeting the N and F genes, capable of detecting as few as 10 genomic copies of PPRV and successfully identifying all four viral lineages [18,19]. Similarly, Kwiatek et al. established an RT-qPCR method based on the N gene, achieving a detection limit of approximately 32 genomic copies [20]. In another study, Polci designed primers and a probe targeting the N gene, which detected around 20 viral copies PPRV [21]. Although these detection methods have high analytical sensitivity, their procedures still require professional laboratory settings and cannot be performed for rapid testing. Early detection of the virus is key to blocking PPR transmission; as a user-friendly detection method, MIRA is increasingly being applied in rapid testing for major animal diseases. Under extreme conditions, the entire reaction process can be completed using only a water bath, and the results can be observed under blue light or combined with LFD for direct visual interpretation. Furthermore, multiple studies have confirmed that, with optimized primers, probes, and experimental conditions, its analytical sensitivity is comparable to that of RT-qPCR. A MIRA assay established by Cui could detect 21.3 copies of chicken chaphamaparvovirus at room temperature within 15 min [22]. A multiplex MIRA method was established, which could detect PEDV and PoRVA with one reaction system [23]. Zhu established an MIRA method for detecting goose astrovirus II, with sensitivity reaching as low as one copy, even surpassing that of RT-qPCR [24]. In summary, this newly developed detection method offers significant advantages for on-site diagnosis of animal diseases due to its convenience, sensitivity, and rapidity.

PPR is a major animal disease with a morbidity rate of 50–90% and a mortality rate that can reach up to 100% [25]. According to relevant data, approximately 80% of the global population of goats and sheep are threatened by PPR. Most of the affected regions are developing countries. The trade of live animals and animal products from affected countries is subject to international restrictions, which has a significant impact on nations and regions where livestock farming is a pillar industry. It is estimated that PPR causes direct and indirect economic losses ranging from USD 1.6 to 2.1 billion annually [26]. Therefore, the application of MIRA—a straightforward, efficient, and user-friendly technology—to the rapid detection of PPR holds significant promise for supporting global PPR control efforts and mitigating its associated economic losses. After screening and optimization, we have established a MIRA-based detection method capable of rapidly identifying all lineages of PPRV. This implies that the method is deployable in any PPR-affected country or region worldwide. All results indicate that the method features good sensitivity, specificity, reproducibility, a short processing time, and easy result interpretation. This significantly reduces the operational difficulty for personnel and enables faster identification of infected animals, allowing for timely intervention to prevent further spread.

As a novel detection method, its operational cost remains higher than that of RT-qPCR, which is a current limitation of this technology. Therefore, reducing its cost is a key issue to be addressed in subsequent research. Nonetheless, MIRA combined with a compact nucleic acid extraction device can serve as a penside method. After preparing the reaction mixture, the entire process can be completed by simply placing the reaction tubes in a constant-temperature water bath or metal bath. Even under extreme conditions, testing can be conducted without the need for a professional laboratory. It remains a highly promising detection technology, playing an important role in supporting the global eradication of PPR.

## Figures and Tables

**Table 1:** Repeatability Test Results.

	Lineage I						Lineage II					
Plasmid copy number copies·μL^−1^	Inter-assay Precision			Intra-assay Precision			Inter-assay Precision			Intra-assay Precision		
	Mean	SD	CV (%)	Mean	SD	CV (%)	Mean	SD	CV (%)	Mean	SD	CV (%)
1 × 10^5^	13,289	229.89	1.73	12,629	73.05	0.58	19,118	242.47	1.27	18,268	249.06	1.36
1 × 10^4^	13,359	181.50	1.361	13,017	69.95	0.54	18,074	407.39	2.25	16,049	118.82	0.74
1 × 10^3^	10,424	162.46	1.56	9214	169.08	1.84	10,168	72.68	0.71	11,182	86.73	0.78
1 × 10^2^	4462	90.48	2.03	4030	43.87	1.09	6429	42.16	0.66	5558	107.804	1.94
1 × 10^1^	1204	33.88	2.81	1108	15.58	1.41	1465	35.86	2.45	1335	14.63	1.10
	Lineage III						Lineage IV					
Plasmid copy number copies·μL^−1^	Inter-assay Precision			Intra-assay Precision			Inter-assay Precision			Intra-assay Precision		
	Mean	SD	CV (%)	Mean	SD	CV (%)	Mean	SD	CV (%)	Mean	SD	CV (%)
1 × 10^5^	17,414	337.50	1.94	16,635	367.52	2.21	18,656	493.79	2.65	19,254	390.20	2.03
1 × 10^4^	16,401	387.00	2.361	15,649	72.58	0.46	17,341	326.09	1.88	17,207	421.45	2.45
1 × 10^3^	11,367	197.14	1.73	9225	73.52	0.80	13,934	251.66	1.81	13,907	206.80	1.49
1 × 10^2^	3476	47.85	1.38	4768	38.21	0.80	9992	277.75	2.78	9070	94.154	1.04
1 × 10^1^	1126	19.96	1.77	951	22.76	2.39	4070	94.60	2.32	3844	34.72	0.90

SD, standard deviation; CV, coefficient of variation.

**Table 2:** Information of clinical samples and detective results of RT-qPCR.

Sample ID			Type of Samples			RT-qPCR (Ct Value)		
1	2	3	lymph node	lymph node	lymph node	30.15	27.85	-
4	5	6	swab	lymph node	swab	-	31.65	29.51
7	8	9	lymph node	lymph node	bronchus	19.58	-	18.07
10	11	12	spleen	lymph node	spleen	15.25	28.38	24.19
13	14	15	lymph node	spleen	swab	-	-	-
16	17	18	swab	swab	lymph node	26.48	-	18.96
19	20	21	lung	lung	bronchus	28.87	-	22.32
22	23	24	swab	lymph node	lymph node	28.78	-	-
25	26	27	lung	rumen	lymph node	-	-	24.26
28	29	30	spleen	swab	swab	-	-	25.78
31	32	33	swab	swab	swab	30.25	32.57	28.78
34	35	36	spleen	lymph node	lymph node	-	-	-
37	38	39	bronchus	bronchus	lymph node	27.85	-	14.02
40	41	42	spleen	lymph node	lymph node	28.05	-	32.05
43	44	45	bronchus	lymph node	swab	33.25	-	21.09
46	47	48	swab	spleen	Lung	-	33.01	35.08

## Data Availability

The original contributions presented in this study are included in the article/Supplementary Material. Further inquiries can be directed to the corresponding author.

## Supplementary Materials

The following supporting information can be downloaded at: https://www.mdpi.com/article/10.3390/vetsci13010110/s1, Table S1: Details of reference strains used in this study.
